# Effects of cyclic-nucleotide derivatives on the growth of human colonic carcinoma xenografts and on cell production in the rat colonic crypt epithelium.

**DOI:** 10.1038/bjc.1981.169

**Published:** 1981-08

**Authors:** P. J. Tutton, D. H. Barkla

## Abstract

Previous studies have shown that various amine hormones are able to influence the growth rate of human colorectal carcinomas propagated as xenografts in immune-deprived mice, and it is now well known that the effects of many amine and other hormones are mediated by cyclic nucleotides, acting as second messengers within cells. In the present study the influence of various derivatives of cyclic adenosine monophosphate and cyclic guanosine monophosphate on the growth of two different lines of colorectal cancer growing in immune-deprived mice, and on the cell production rate in the colonic crypt epithelium of the rat, was assessed. Growth of each tumour line, as well as crypt-cell production, was suppressed by treatment wit N6O2' dibutyryl and N6 monobutyryl derivatives of cyclic adenosine monophosphate. Dibutyryl cyclic guanosine monophosphate, on the other hand, was found to promote the growth of Tumour HXK4 and to promote crypt cell production, but to have no significant effect on Tumour HXM2.


*Br. J. Cancer* (1981) **44**, 182

### EFFECTS OF CYCLIC-NUCLEOTIDE DERIVATIVES ON THE GROWTH OF HUMAN COLONIC CARCINOMA XENOGRAFTS AND ON CELL PRODUCTION IN THE RAT COLONIC CRYPT EPITHELIUM

P. J. M. TUTTON AND D. H. BARKLA

*From the Department of Anatomy, Monash University, Victoria, Australia*

Received 13 January 1981 Accepted 24 April 1981

**Summary.—Previous studies have shown that various amine hormones are able to influence the growth rate of human colorectal carcinomas propagated as xenografts in immune-deprived mice, and it is now well known that the effects of many amine and other hormones are mediated by cyclic nucleotides, acting as second messengers within cells. In the present study the influence of various derivatives of cyclic adenosine monophosphate and cyclic guanosine monophosphate on the growth of two different lines of colorectal cancer growing in immune-deprived mice, and on the cell production rate in the colonic crypt epithelium of the rat, was assessed. Growth of each tumour line, as well as crypt-cell production, was suppressed by treatment with $N^6O^{2'}$ dibutyryl and $N^6$ monobutyryl derivatives of cyclic adenosine monophosphate. Dibutyryl cyclic guanosine monophosphate, on the other hand, was found to promote the growth of Tumour HXK4 and to promote crypt cell production, but to have no significant effect on Tumour HXM2.**

THERE IS MOUNTING EVIDENCE that cell proliferation in the colonic crypt epithelium and in colonic tumours is influenced by amine hormones. Adrenaline and other beta-adrenergic agonists have been shown to inhibit cell division in dimethylhydrazine-induced tumours of rat colon (Tutton & Barkla, 1977*a*), whereas histamine (acting *via* histamine-$H_2$ receptors) and serotonin both promote cell division in these tumours (Tutton & Barkla, 1978*a*, *b*; 1980*a*). By contrast noradrenaline, acting *via* an $\alpha$-adrenergic receptor, promotes cell production in the colonic crypt epithelium (Tutton & Barkla, 1977*a*; Tutton & Steel, 1979) have shown that the growth rate of human colonic tumours propagated as xenografts in immune-deprived mice is also influenced by amine hormones. Growth of one tumour line, HXK4, a moderately to well differentiated colonic adenocarcinoma (Nowak *et al.*, 1978) was inhibited by the histamine-$H_2$-receptor antagonist, cimetidine, whereas another colonic tumour, HXK7 (a moderately to poorly differentiated colonic adenocarcinoma) was inhibited by an anti-serotoninergic drug, BW501c. The growth of Tumour HXK4 was also inhibited by adrenaline acting through a $\beta$-adrenoceptor.

Cyclic nucleotides have been implicated as intracellular mediators of the effects of many hormones, including the biogenic amines adrenaline, noradrenaline, histamine and serotonin. The catecholamines, such as adrenaline and noradrenaline, have been shown to act *via* receptors which were originally classified into 2 groups, $\alpha$ and $\beta$ (Ahlquist, 1948) and which have subsequently been sub-classified into 4 groups, $\alpha_1$ and $\alpha_2$ (Berthelson & Pettinger, 1977), and $\beta_1$ and $\beta_2$ (Lands *et al.*, 1967). The response to $\alpha_1$ receptors is mediated *via* calcium ions, whereas that of alpha$_2$ receptors is mediated by inhibi-

Correspondence: Dr P. J. M. Tutton, Department of Anatomy, Faculty of Medicine, Monash University, Clayton, Victoria 3168, Australia.

tion of adenylate cyclases and consequent lowering of intracellular cyclic AMP level (Fain & Garcia-Sainz, 1980). The response to both types of $\beta$ adrenoceptors is mediated by activation of adenylate cyclase and consequent elevation of cellular cyclic AMP levels (Maguire *et al.*, 1977). The cellular responses to histamine are mediated by receptors which have been termed H1 and H2 (Ash & Schild, 1966; Black *et al.*, 1972) with $H_1$-receptor effects being mediated by calcium ions and $H_2$-receptor effects being mediated by cyclic AMP (Fain & Garcia-Sainz, 1980). The effects of serotonin have been attributed to raised cyclic AMP levels in some tissues (Enjalbert *et al.*, 1978) and to raised cyclic GMP levels in other tissues (Goldberg & Haddox, 1977).

The effects of amine hormones on xenograft growth are not readily explained in terms of cyclic nucleotide actions, for two reasons. First, in Tumour Line HXK4, adrenaline (acting through a $\beta$-adrenoceptor and hence presumably cAMP-linked) and histamine (acting through a histamine-$H_2$ receptor, also presumably cAMP-linked) have opposing actions on tumour growth rate. Secondly, growth appears to be promoted in one tumour line (HXK4) by a cAMP-linked agent (histamine *via* $H_2$ receptors) and in the other line (HXK7) by an agent (serotonin) which is possibly a cGMP-linked agent. In an attempt to resolve these apparent inconsistencies, the effect of various derivatives of cAMP and cGMP on the growth of a histamine-dependent line and a serotonin-dependent line of colonic tumours, and on cell production in the colonic crypt epithelium, was examined.

### MATERIALS AND METHODS

*Estimation of the mitotic rate in the colonic crypt epithelium.*—Male Sprague–Dawley rats were fed Clark No. 2 pellets, tap water *ad libitum*, and housed at 21–24°C with artificial light from 07:00 to 21:00 and darkness from 21:00 to 07:00. Animals were given vinblastine sulphate (Velbe, Elly Lilly and Co., Indianapolis, U.S.A.) at a dose of 4 mg/kg by i.p. injection at 12:00 and were killed by decapitation between 12:45 and 16:00. This dose of vinblastine was found, in preliminary experiments, to be the lowest that provides reliable metaphase arrest in the colonic crypts. Counts of metaphase and non-metaphase cells in histological sections in crypts of Lieberkuhn of the descending colon were made as previously described (Tutton & Barkla, 1976). All metaphase indices were then corrected for the differences in size between metaphase and non-metaphase nuclei and for the geometric artefact described by Tannock (1967). Details of these correction factors are described elsewhere.

Graphs of corrected metaphase index *vs* duration of vinblastine treatment were then constructed for each experimental group of tissues with mitoses blocked for 0·75–4 h. The least-squares estimate of the regression coefficient for each of the graphs was then calculated; this value represents the rate at which cells enter metaphase and has the units of mitoses/cell/h. The statistical significance of apparent differences between regression coefficients for different experimental groups of tissue was then estimated by analysis of variance (Bliss, 1967).

Initially, cell proliferation was studied in the colonic crypts of 14 normal rats.

Additional groups of 6 rats were treated with $N^6O^{2'}$ dibutyryl, $N^6$ monobutyryl, $O^{2'}$ monobutyryl and 8-bromo cyclic AMP or $N^6O^{2'}$ dibutyryl cyclic GMP, each at a dose of 2 mg/kg injected i.p. All cyclic-nucleotide derivatives were purchased from the Sigma Chemical Company, St Louis, U.S.A. A further group of 6 rats was treated with sodium butyrate at a dose of 7 mg/kg. This dose was used since it represents the amount of butyrate moiety that was contained in the highest dose of dibutyryl cAMP.

*Xenograft technique.*—Male and female CBA/lac mice were bred in our laboratory and immune-deprived by a technique similar to that reported by Steel *et al.* (1978). Mice aged 16–20 days were thymectomized under ketamine anaesthesia (Ketlar, 0·15 mg/kg i.p.). After an interval of 18–21 days the mice were injected with cytosine arabinoside (Cytosar, the Upjohn Company) as a dose of 200 mg/kg i.p. and then subjected, 48 h later, to 8·5 gray of whole-body irradiation from an X-ray source at 180 keV. Pre-treatment with Cytosar obviated the need for marrow reconstitution that was previously necessary

(Lammerton & Steel, 1975). Small fragments of tumour, 2–3 mm in greatest linear dimensions, were implanted in the flanks of mice using ketamine anaesthesia. All implantations were undertaken in a Biohazard Cabinet (Clemco, Australia, Ltd.). Tumour Line HXK4, a moderately to well differentiated adenocarcinoma, originally described by Nowak *et al.* (1978), was obtained from the Institute of Cancer Research, Royal Marsden Hospital (U.K.). This tumour line had previously been assessed as histamine-dependent, in the sense that its growth in immune-deprived mice was inhibited by administration of the histamine-$H_2$-receptor antagonist Cimetidine (Tutton & Steel, 1979). Tumour Line HXM2 was established by the authors from a surgically resected specimen of descending colon tumour. This tumour was histologically poorly differentiated and assessed as being serotonin-dependent, in the sense that its growth was inhibited by the antiserotoninergic drug BW501c (Tutton & Barkla, unpublished).

*Tumour measurement.*—Starting on the 24th to 28th day after implantation, tumours were measured every 1 or 2 days. The greatest and least superficial diameters were measured, using vernier calipers, and the xenograft volume calculated by the formula (mean diameter)$^3 \times \Pi/6$. The volume of each tumour after $t$ days of assessment was divided by the volume of the same tumour at the start of assessment ($V_0$) to obtain the relative tumour volume ($V_t/V_0$). The mean of the logarithm of this quotient was then plotted as a function of time for each treatment group of mice. The relative tumour volume was calculated because inter-xenograft variations in this parameter arise only during treatment; the logarithm of the quotient was plotted against time because of the previously observed linearity of log volume *vs* time for xenografts (Lamerton & Steel, 1975). Tumour volume-doubling times for control tumours were calculated from the linear regression of log relative tumour volume *vs* time. The statistical significance of any apparent difference between the relative volumes of various groups of xenografts at a particular time after the start of treatment was assessed using the Mann–Whitney, non-parametric test for ranked observations (Sokal & Rohlf, 1969). The control group of tumours for Line HXK4 contained 16 xenografts and the control group of tumours for Line HXM2 contained 17 xenografts. Each experimental group consisted of 10–16 xenografts. Mice were treated with $N^6O^{2'}$ dibutyryl, $N^6$ monobutyryl, $O^{2'}$ monobutyryl or 8-bromo cyclic AMP at doses of either 2·0 or 20 mg/kg, $N^6O^{2'}$ dibutyryl cyclic GMP at doses of either 0·2 or 2 mg/kg, or a combination of dibutyryl cyclic AMP (20 mg/kg) and a phosphodiesterase inhibitor, papaverine HCl at a dose of 50 mg/kg (Amer & Kreighbaum, 1975). An additional group of mice bearing Tumour HXK4 was treated with sodium butyrate at a dose of 7 mg/kg. All mice were treated twice daily.

### RESULTS

The influence of various cyclic nucleotide derivatives and of sodium butyrate on the colonic crypt-cell production rate is shown in the Table.

TABLE.—*The influence of cyclic-nucleotide derivatives on colonic crypt-cell proliferation*

| Treatment | Dose (mg/kg) | Mitotic rate—mean ± s.e. (mitosis/cell/h) | *P* (*vs* control) |
|---|---|---|---|
| Nil | — | 0·024 ± 0·006 | — |
| Sodium butyrate | 7·0 | 0·040 ± 0·004 | NS |
| Dibutyryl cGMP | 2·0 | 0·035 ± 0·006 | NS |
| | 0·2 | 0·063 ± 0·012 | < 0·001 |
| | 0·02 | 0·057 ± 0·010 | < 0·01 |
| Dibutyryl cAMP | 20 | 0·008 ± 0·004 | < 0·01 |
| | 2·0 | 0·009 ± 0·004 | < 0·01 |
| | 0·2 | 0·008 ± 0·007 | < 0·05 |
| $N^6$-monobutyryl cAMP | 2·0 | 0·012 ± 0·002 | < 0·05 |
| $O^{2'}$-monobutyryl cAMP | 2·0 | 0·019 ± 0·003 | NS |
| 8-Bromo cAMP | 2·0 | 0·036 ± 0·007 | NS |

Growth curves for Tumour HXK4 are illustrated in Figs 1, 2 and 3, and those for HXM2 in Figs 4 and 5. Tumour HXK4 had a mean doubling time of 8·1 days and HXM2 had a mean doubling time of 7·3 days. In each case db cAMP was seen to inhibit xenograft growth, though in the case of HXK4 using a dose of 20 mg/kg, and HXM2 using a dose of 2 mg/kg, this response was short-lived. Of the other cAMP derivatives tested, $N^6$ monobutyryl cAMP was of similar effectiveness to db cAMP, whereas other derivatives were marginally effective or ineffective. In HXK4 tumours treated with db cAMP and the phosphodiesterase inhibitor, papaverine HCl, growth remained inhibited for 5 days and, in HXM2 tumours treated with db cAMP alone at a dose of 20 mg/kg, growth remained inhibited for 6 days, and the mean tumour volume after 8 days of treatment was similar to that at the beginning of the experiment. Papaverine alone also inhibited the growth of Tumour HXK4, but was less effective than db cAMP alone. Treatment with db cGMP at a dose of 2 mg/kg had a short-term stimulatory effect on Tumour HXK4 and no statistically significant effect on tumour HXM2.

FIG. 1.—Log relative tumour volume *vs* days of treatment for Tumour Line HXK4. ——, control; – – –, dibutyryl cGMP (2 mg/kg).

FIG. 2.—Log relative tumour volume *vs* days of treatment for Tumour Line HXK4; ——, control; - - - -, sodium butyrate (7 mg/kg); – – –, dibutyryl cAMP (20 mg/kg); ·····, papaverine HCl (50 mg/kg); ·–·–·, dibutyryl cAMP and papaverine HCl.

FIG. 3.—Log relative tumour volume *vs* days of treatment for Tumour Line HXK4; ——, control; - - - -, dibutyryl cAMP; ·–·–·, $N^6$-monobutyryl cAMP; – – –, $O^{2'}$-monobutyryl cAMP; ·····, 8-bromo cAMP. All doses 2 mg/kg.

FIG. 4.—Log relative tumour volume *vs* days of treatment for Tumour Line HXM2; ——, control; – – –, dibutyryl cGMP (2 mg/kg).

FIG. 5.—Log relative tumour volume *vs* days of treatment for Tumour Line HXM2; ——, control; ·····, dibutyryl cAMP (2·0 mg/kg); – – –, dibutyryl cAMP (20 mg/kg).

DISCUSSION

The observed inhibitory effect of cAMP derivatives on the growth of both lines of human colonic tumour resembles the effects of this agent on the mitotic rate in rat primary colonic tumours (Tutton & Barkla, 1980*c*) and the stimulating effect of db cGMP on Tumour Line HXK4 also resembles the effect of this agent on rat tumours (Tutton & Barkla, 1980*b*). Since the growth of Tumour Line HXK4 is inhibited by cAMP derivatives and promoted by cGMP derivatives, it would appear unlikely that the previously reported growth promoting effect of histamine on this tumour is mediated by cAMP; in fact these results raise the possibility that the effects of histamine-$H_2$-receptor agonism is mediated by cGMP.

The Yin Yang hypothesis (Goldberg *et al.*, 1974), which proposes that various biological processes, including cell division, are controlled by the molar ratio of cyclic GMP to cyclic AMP, is not, however, uniformly supported by data from our laboratory. We have reported previously that in the jejunal crypt epithelium significant inhibition of cell proliferation by db cAMP was not obtained with doses between 0·2 and 20 mg/kg (Tutton & Barkla, 1980*c*). It must be admitted that in these experiments direct inhibition by cAMP may have been opposed by indirect stimulation by increased circulating levels of thyroid hormones, which have previously been shown to promote jejunal crypt-cell proliferation (Carriere, 1976; Tutton, 1965). Secretion of glucocorticoids may also be increased by treatment with cAMP derivatives (Perlmutter *et al.*, 1971) but this would not appear to explain the present results, since glucocorticoids themselves appear not to influence colonic crypt-cell proliferation, and to promote rather than inhibit cell proliferation in colonic tumours (Tutton & Barkla, submitted for publication). Clearly, the suggested role of cyclic nucleotides as intracellular mediators for the amine hormones which appear to influence intestinal epithelial-cell proliferation needs to be confirmed using radioimmunoassay and possibly quantitative immunocytochemistry of cyclic nucleotides in amine-treated and control tissues.

If cyclic GMP is a growth stimulant in colonic tumours and a mitogen in both colonic tumours and in the colonic crypt epithelium (Tutton & Barkla, 1980*b*), has the metabolism of biogenic amines and cyclic nucleotides any relevance to the basic biology of neoplastic change? At present the only optimistic lead to this question lies in the reported differences between particulate (membrane-bound) and soluble (cytoplasmic) forms of guanylate cyclase in various tissues (Kimura & Murad, 1975). Exposure of the colonic epithelium to the carcinogen N-methyl-nitro-nitrosoguanidine results in a shift from particulate to soluble guanylate cyclase (de Rubertis & Craven, 1977). Stimulation of soluble guanylate cyclase by biogenic amines requires the cells to have either an amine-uptake process or an amine-regulated calcium-gating uptake (Fain & Garcia-Sainz, 1980). Evidence for such an amine-uptake process in neoplastic but not in normal colonic epithelial cells has been reported; toxic analogues of serotonin, (5,6- and 5,7-dihydroxytryptamine) cause rapid cytoplasmic changes in DMH-induced colonic tumour cells, but not in jejunal crypt cells (Tutton & Barkla, 1977*b*; 1979). The biological significance of amine uptake by tumour cells remains to be assessed.

Whatever differences exist between the mechanism controlling the synthesis and degradation of cyclic nucleotides in normal and malignant tissues, the fact remains that cellular levels of cGMP are higher, and levels of cAMP are lower in colonic tumours than in colonic mucosa (de Rubertis *et al.*, 1976). In addition, plasma and urinary levels of cyclic GMP are raised in patients with disseminated bowel cancer (Chawla *et al.*, 1979) and pharmacokinetic studies have shown that this increase in plasma and urinary levels of cGMP is due to a substantial increase in its production (Murray *et al.*, 1979). These cyclic-nucleotide assay results, seen in the light of our cell-kinetic results, suggest that cyclic nucleotides may have an exceedingly important role in controlling tumour growth.

This work was done during the tenure of a research grant awarded by the Anti-Cancer Council of Victoria. Skilled technical assistance was provided by Fiona Christensen and Fiona McCready.

### REFERENCES

AHLQUIST, R. P. (1948) A study of adrenotropic receptors. *Am. J. Physiol.*, **153**, 586.

AMER, A. S. & KREIGHBAUM, W. E. (1975) Cyclic nucleotide phosphodiesterases: Properties, activations, inhibitors, structure–activity relationships and possible role in drug development. *J. Pharmac. Sci.*, **64**, 1.

ASH, A. S. F. & SCHILD, H. D. (1966) Receptors mediating some actions of histamine. *Br. J. Pharmacol. Chemother.*, **27**, 427.

BERTHELSON, S. & PETTINGER, W. A. (1977) A functional basis for classification of α-adrenergic receptors. *Life Sci.*, **21**, 595.

BLACK, J. W., DUNCAN, W. A. M., DURRANT, C. J., GANELLIN, C. R. & PARSONS, E. M. (1972) Definition and antagonism of histamine $H_2$-receptors. *Nature*, **236**, 385.

BLISS, C. I. (1967) *Statistics in Biology*, Vol. I. New York: McGraw-Hill. p. 420.

CARRIERE, R. (1965) The influence of thyroid and testicular hormones on the epithelium of crypts of Lieberkuhn in the rat's intestine. *Anat. Rec.*, **156**, 423.

CHAWLA, R. K., NOXIN, D. W., SHOJI, M. & RUDMAN, D. (1979) Plasma and urinary cyclic guanosine 3′:5′-monophosphate in disseminated cancer. *Ann. Intern. Med.*, **91**, 862.

DE RUBERTIS, F. R., CHAYOTH, R. & FIELD, J. B. (1976) The content and metabolism of cyclic adenosine 3′5′-monophosphate and cyclic guanosine 3′,5′-monophosphate in adenocarcinoma of the human colon. *J. Clin. Invest.*, **57**, 641.

DE RUBERTIS, F. R. & CRAVEN, P. A. (1977) Activation of the guanylate cyclase-guanosine 3′5′-monophosphate system of colonic mucosa by N-methyl-N′-nitrosoguanidine. *Cancer*, **40**, 2600.

ENJALBERT, A., BOURGOIN, S., HAMON, M., ADRIEN, J. & BOCKAERT, J. (1978) Postsynaptic serotonin-sensitive adenylate cyclase in the central nervous system. *Mol. Pharmacol.*, **14**, 2.

FAIN, J. N. & GARCIA-SAINZ, J. A. (1980) Role of phosphatidylinositol turnover in $alpha_1$, and of adenylate cyclase inhibition in $alpha_2$ effects of catecholamines. *Life Sci.*, **26**, 1183.

GOLDBERG, N. G. & HADDOX, M. K. (1977) Cyclic GMP metabolism and involvement in biological regulation. *Ann. Rev. Biochem.*, **46**, 823.

GOLDBERG, N. D., HADDOX, M. K., DUNHAM, E., LOPEZ, C. & HADDON, J. W. (1974) The yin yang hypothesis of biological control: Opposing influences of cyclic GMP and cyclic AMP in the regulation of cell proliferation and other biological processes. In *The Cold Spring Harbour Symposium on the Regulation of Proliferation in Animal Cells* (Eds Clarkson & Baserga). New York: Cold Spring Harbour Labs.

KIMURA, H. & MURAD, F. (1975) Two forms of guanylate cyclase in mammalian tissues and possible mechanisms for their regulation. *Metabolism*, **24**, 439.

LAMERTON, L. F. & STEEL, G. G. (1975) Growth kinetics of human large bowel cancer growing in immune-deprived mice and some chemotherapeutic observations. *Cancer*, **36**, 2431.

LANDS, A. M., ARNOLD, A., MCAULIFF, J. P., LUDENA, F. P. & BROWN, T. G. (1967) Differentiation of receptor systems activated by sympathomimetic amines. *Nature*, **214**, 597.

MAGUIRE, M. E., ROSS, E. M. & GILMAN, A. G. (1977) β-Adrenergic receptor: Ligand binding studies and interaction with adenylate cyclase. *Adv. Cyclic Nuc. Res.*, **8**, 1.

MURRAY, T., LAWSON, D., CHAWLA, R. K. & RUDMAN, D. (1979) Elevated plasma and urinary cyclic GMP and increased production rate in patients with neoplastic disease. *Clin. Res.*, **27**, 759a.

NOWAK, K., PECKHAM, M. J. & STEEL, G. G. (1978) Variations in response of xenografts of colo-rectal carcinoma to chemotherapy. *Br. J. Cancer*, **37**, 576.

PERLMUTTER, F., RAPINO, E. & SAFFRON, M. (1971) ACTH and cyclic adenine nucleotides do not evoke identical adrenocortical responses. *Endocrinology*, **89**, 963.

SOKAL, R. R. & ROHLF, F. J. (1969) *Biometry*. San Francisco: W. H. Freeman.

STEEL, G. G., COURTENAY, V. D. & ROSTOM, A. Y. (1978) Improved immune-suppression techniques for xenografting human tumours. *Br. J. Cancer*, **32**, 224.

TANNOCK, I. F. (1967) A comparison of the relative efficiencies of various metaphase arrest agents. *Exp. Cell Res.*, **47**, 345.

TUTTON, P. J. M. (1976) The influence of thyroidectomy and triiodothyronine administration on epithelial cell proliferation in the jejunum of rat. *Virchows Arch.* [*Cell Pathol.*], **20**, 139.

TUTTON, P. J. M. & BARKLA, D. H. (1976) Cell proliferation in the descending colon of dimethylhydrazine-treated rats and in dimethylhydrazine-induced adenocarcinoma. *Virchows Arch.* [*Cell Pathol.*], **21**, 147.

TUTTON, P. J. M. & BARKLA, D. H. (1977*a*) The influence of adrenoceptor activity on cell proliferation in colonic crypt epithelium and in colonic adenocarcinomata. *Virchows Arch.* [*Cell Pathol.*], **24**, 139.

TUTTON, P. J. M. & BARKLA, D. H. (1977*b*) Cytotoxicity of 5,6-dihydroxytryptamine in dimethylhydrazine-induced carcinomas of rat colon. *Cancer Res.*, **37**, 1241.

TUTTON, P. J. M. & BARKLA, D. H. (1978*a*) Stimulation of cell proliferation by histamine $H_2$-receptors in dimethylhydrazine-induced adenocarcinomata. *Cell Biol. Int. Rep.*, **2**, 199.

TUTTON, P. J. M. & BARKLA, D. H. (1978*b*) The influence of serotonin on the mitotic rate in the colonic crypt epithelium and in colonic adenocarcinoma in rats. *Clin. Exp. Pharmacol. Physiol.*, **5**, 91.

TUTTON, P. J. M. & BARKLA, D. H. (1979) Evaluation of the cytotoxicity of dihydroxytryptamines and 5-hydroxytryptamine antagonists as cytotoxic agents in dimethylhydrazine-induced adenocarcinomata. *Cancer Chemother. Pharmacol.*, ,**1** 209.

TUTTON, P. J. M. & BARKLA, D. H. (1980*a*) Neural control of colonic cell proliferation. *Cancer*, **45**, 1172.

TUTTON, P. M. J. & BARKLA, D. H. (1980*b*) A final common pathway promoting cell proliferation in normal and in neoplastic intestinal epithelia. In *Cell Proliferation in the Gastrointestinal Tract.* Ed. Appleton. London: Pitman Medical. p. 298.

TUTRON, P. J. M. & BARKLA, D. H. (1980*c*) The influence of dibutyryl adenosine cyclic monophosphate on cell proliferation in the epithelium of the jejunal crypts, the colonic crypts and in colonic carcinomata of rat. *Clin. Exp. Pharmacol. Physiol.*, **7**, 275.

TUTTON, P. J. M. & STEEL, G. G. (1979) Influence of biogenic amines on the growth of xenografted human colorectal carcinomas. *Br. J. Cancer*, **40**, 743.


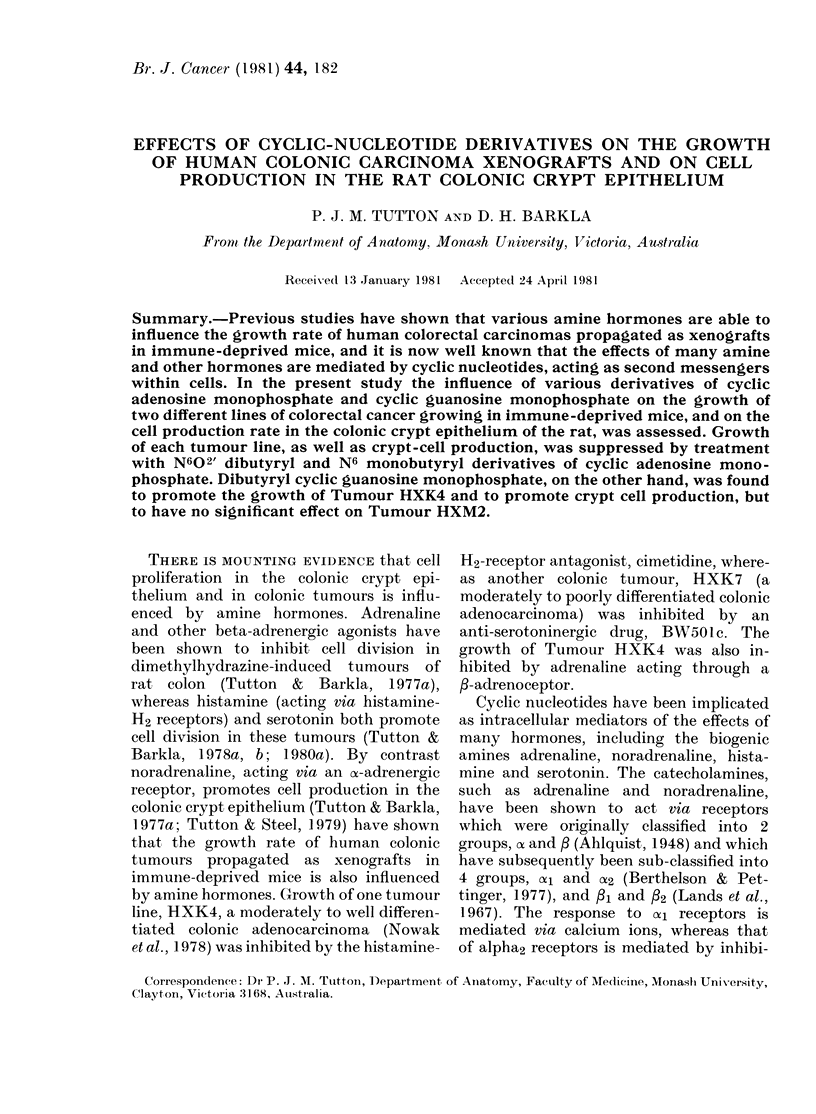

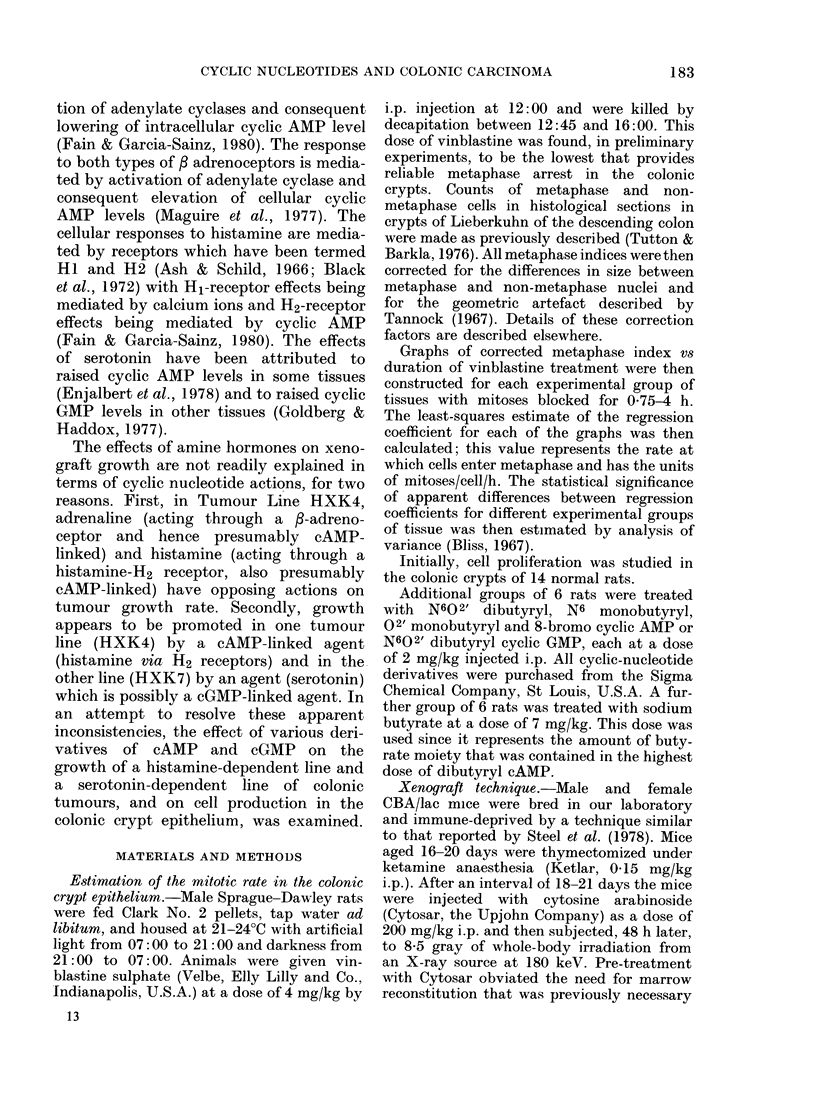

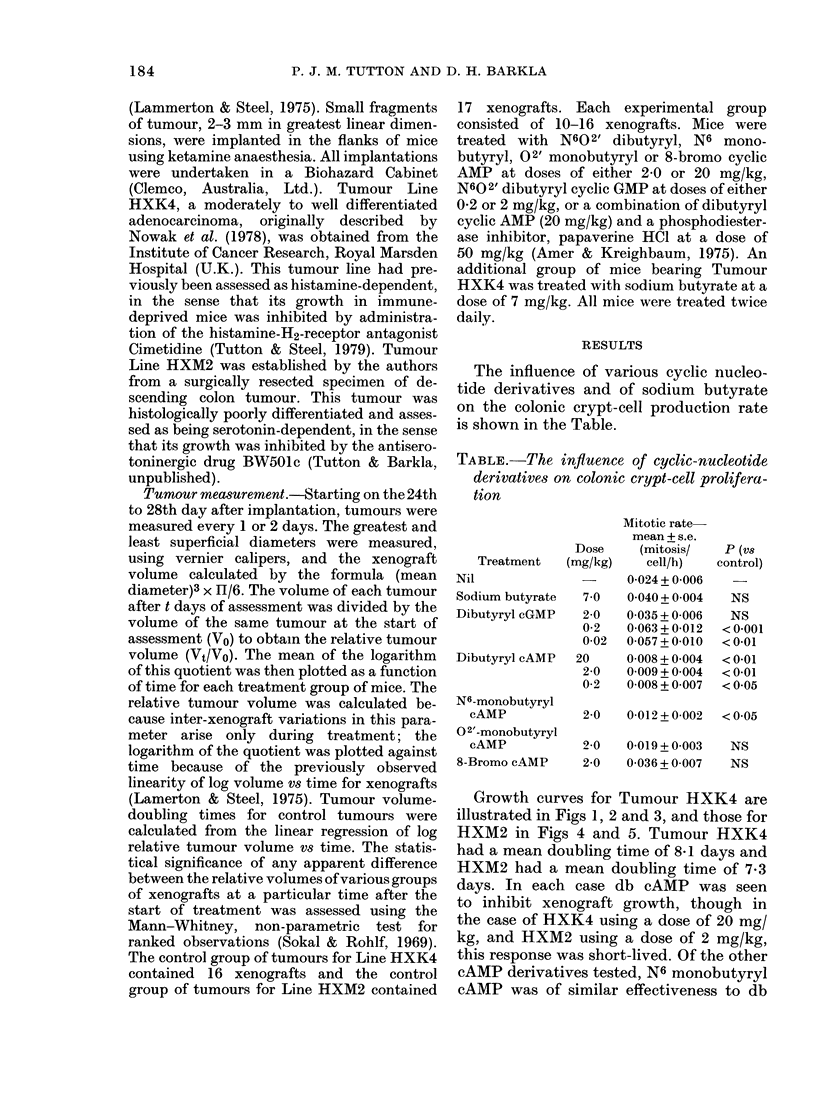

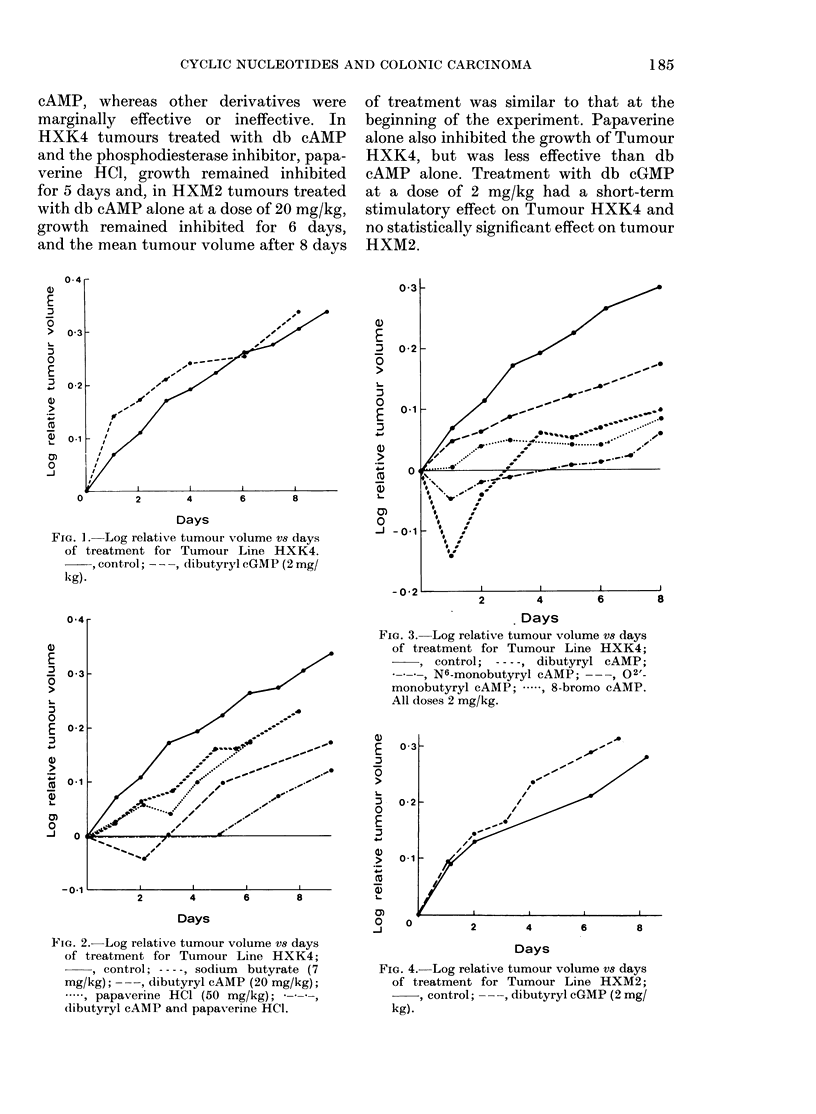

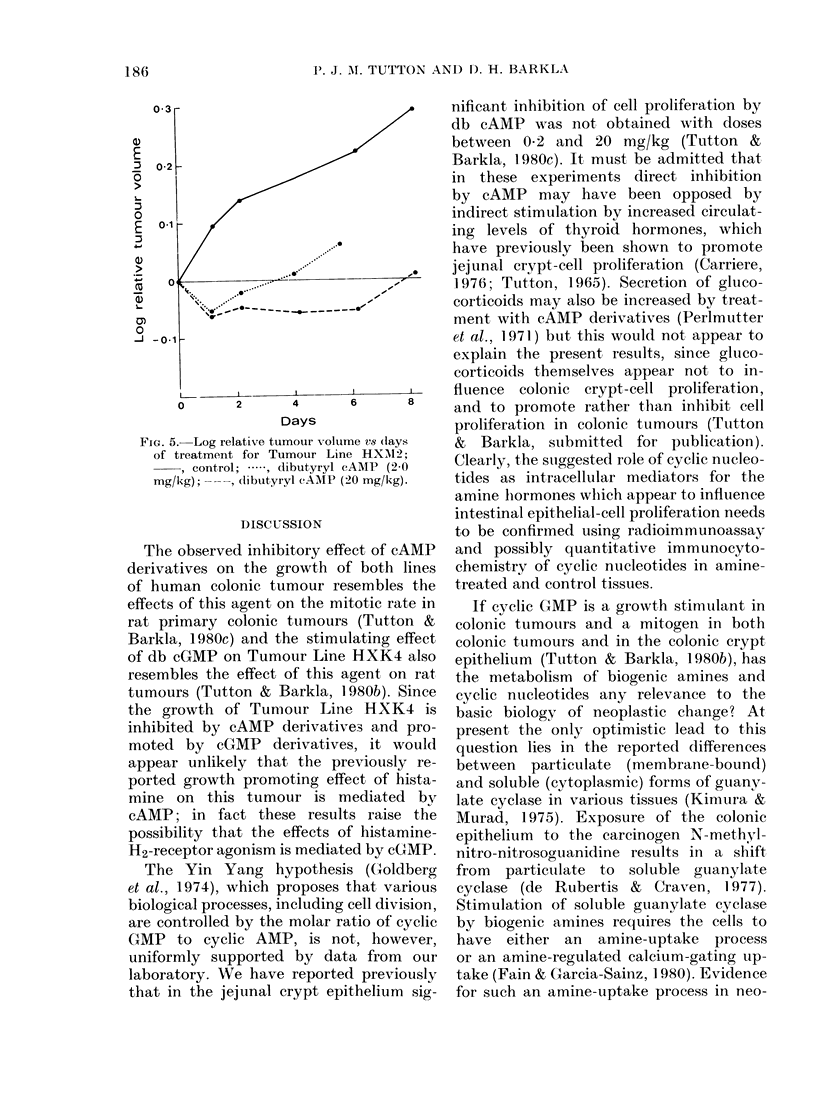

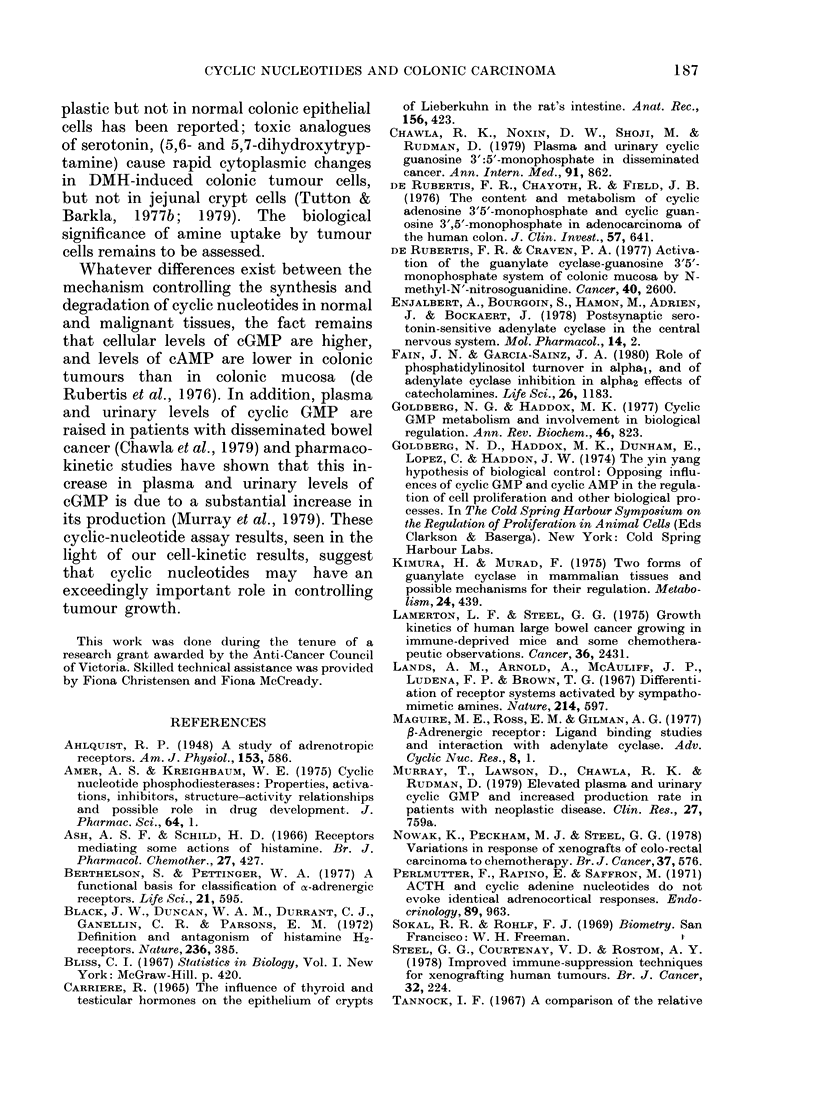

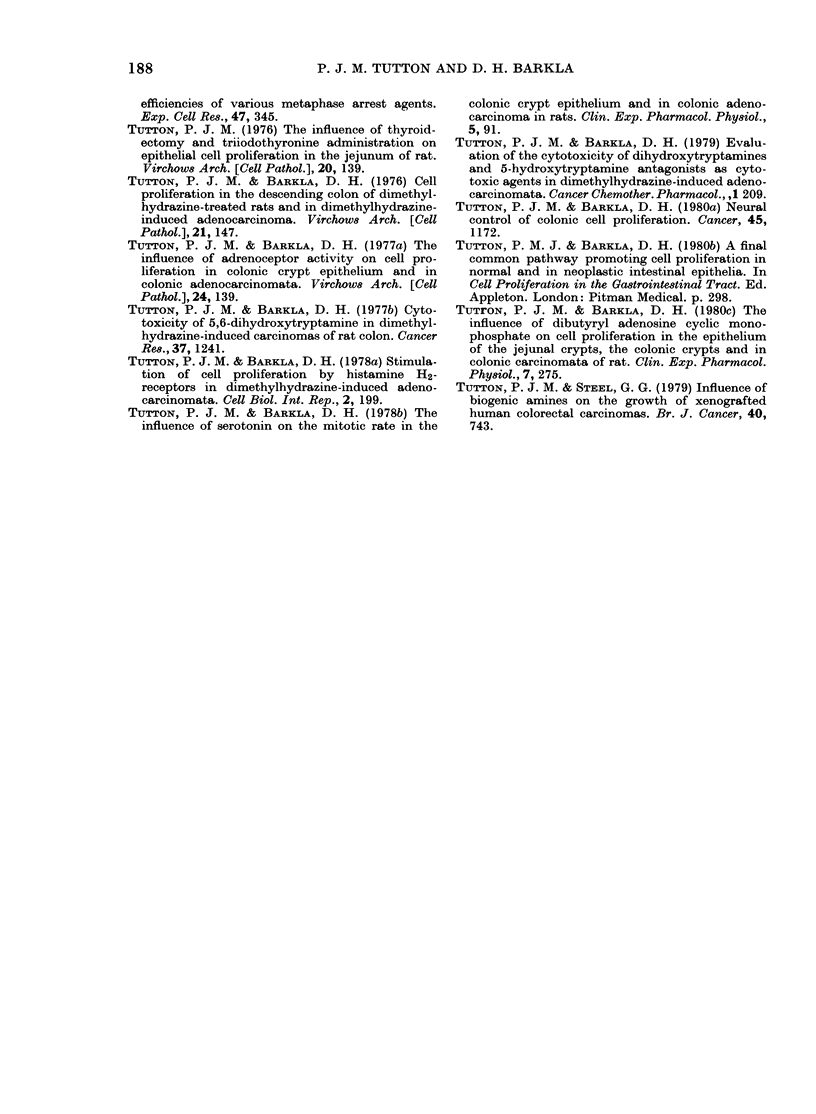


## References

[OCR_00668] Ash A. S., Schild H. O. (1966). Receptors mediating some actions of histamine.. Br J Pharmacol Chemother.

[OCR_00673] Berthelsen S., Pettinger W. A. (1977). A functional basis for classification of alpha-adrenergic receptors.. Life Sci.

[OCR_00678] Black J. W., Duncan W. A., Durant C. J., Ganellin C. R., Parsons E. M. (1972). Definition and antagonism of histamine H 2 -receptors.. Nature.

[OCR_00688] Carriere R. M. (1966). The influence of thyroid and testicular hormones on the epithelium of crypts of Lieberkühn in the rat's intestine.. Anat Rec.

[OCR_00695] Chawla R. K., Nixon D. W., Shoji M., Rudman D. (1979). Plasma and urine cyclic guanosine 3':5'-monophosphate in disseminated cancer.. Ann Intern Med.

[OCR_00701] DeRubertis F. R., Chayoth R., Field J. B. (1976). The content and metabolism of cyclic adenosine 3', 5'-monophosphate and cyclic guanosine 3', 5'-monophosphate in adenocarcinoma of the human colon.. J Clin Invest.

[OCR_00708] DeRubertis F. R., Craven P. A. (1977). Activation of the guanylate cyclase-guanosine 3'5' monophosphate system of colonic mucosa by n-methyl-n'-nitro-n-nitrosoguanidine.. Cancer.

[OCR_00714] Enjalbert A., Bourgoin S., Hamon M., Adrien J., Bockaert J. (1978). Postsynaptic serotonin-sensitive adenylate cyclase in the central nervous system. I. Development and distribution of serotonin and dopamine-sensitive adenylate cyclases in rat and guinea pig brain.. Mol Pharmacol.

[OCR_00720] Fain J. N., García-Sáinz J. A. (1980). Role of phosphatidylinositol turnover in alpha 1 and of adenylate cyclase inhibition in alpha 2 effects of catecholamines.. Life Sci.

[OCR_00726] Goldberg N. D., Haddox M. K. (1977). Cyclic GMP metabolism and involvement in biological regulation.. Annu Rev Biochem.

[OCR_00742] Kimura H., Murad F. (1975). Two forms of guanylate cyclase in mammalian tissues and possible mechanisms for their regulation.. Metabolism.

[OCR_00748] Lamerton L. F., Steel G. G. (1975). Growth kinetics of human large bowel cancer growing in immune-deprived mice and some chemotherapeutic observations.. Cancer.

[OCR_00754] Lands A. M., Arnold A., McAuliff J. P., Luduena F. P., Brown T. G. (1967). Differentiation of receptor systems activated by sympathomimetic amines.. Nature.

[OCR_00773] Nowak K., Peckham M. J., Steel G. G. (1978). Variation in response of xenografts of colo-rectal carcinoma to chemotherapy.. Br J Cancer.

[OCR_00777] Pearlmutter A. F., Rapino E., Saffran M. (1971). ACTH and cyclic adenine nucleotides do not provoke identical adrenocortical responses.. Endocrinology.

[OCR_00661] Samir Amer M., Kreighbaum W. E. (1975). Cyclic nucleotide phosphodiesterases: properties, activators, inhibitors, structure--activity relationships, and possible role in drug development.. J Pharm Sci.

[OCR_00787] Steel G. G., Courtenay V. D., Rostom A. Y. (1978). Improved immune-suppression techniques for the exongrafting of human tumours.. Br J Cancer.

[OCR_00806] Tutton P. J., Barkla D. H. (1976). Cell proliferation in the descending colon of dimethylhydrazine treated rats and in dimethylhydrazine induced adenocarcinomata.. Virchows Arch B Cell Pathol.

[OCR_00820] Tutton P. J., Barkla D. H. (1977). Cytotoxicity of 5,6-dihydroxytryptamine in dimethylhydrazine-induced carcinomas of rat colon.. Cancer Res.

[OCR_00840] Tutton P. J., Barkla D. H. (1978). Evaluation of the cytotoxicity of dihydroxytryptamines and 5-hydroxytryptamine antagonists as cytotoxic agents in dimethylhydrazine-induced adenocarcinomata.. Cancer Chemother Pharmacol.

[OCR_00846] Tutton P. J., Barkla D. H. (1980). Neural control of colonic cell proliferation.. Cancer.

[OCR_00826] Tutton P. J., Barkla D. H. (1978). Stimulation of cell proliferation by histamine H2 receptors in dimethylhdrazine-induced adenocarcinomata.. Cell Biol Int Rep.

[OCR_00813] Tutton P. J., Barkla D. H. (1977). The influence of adrenoceptor activity on cell proliferation in colonic crypt ipithelium and in colonic adenocarcinomata.. Virchows Arch B Cell Pathol.

[OCR_00858] Tutton P. J., Barkla D. H. (1980). The influence of dibutyryl adenosine cyclic monophosphate on cell proliferation in the epithelium of the jejunal crypts, the colonic crypts and in colonic carcinomata of rat.. Clin Exp Pharmacol Physiol.

[OCR_00832] Tutton P. J., Barkla D. H. (1978). The influence of serotonin on the mitotic rate in the colonic crypt epithelium and in colonic adenocarcinoma in rats.. Clin Exp Pharmacol Physiol.

[OCR_00866] Tutton P. J., Steel G. G. (1979). Influence of biogenic amines on the growth of xenografted human colorectal carcinomas.. Br J Cancer.

[OCR_00802] Tutton P. J. (1976). The influence of thyroidectomy and of triiodothyronine administration on epithelial cell proliferation in the jejunum of rat.. Virchows Arch B Cell Pathol.

